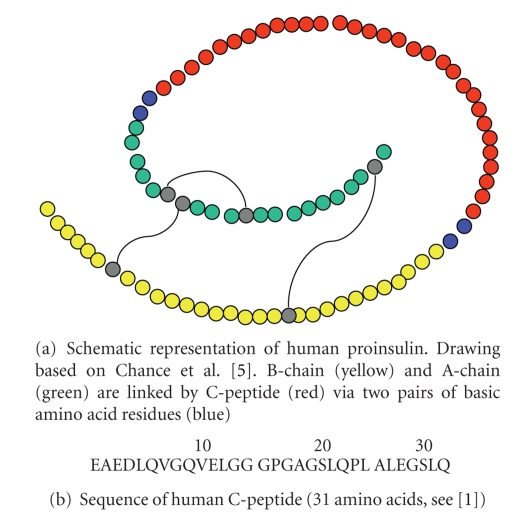# History and Diagnostic Significance of C-Peptide

**DOI:** 10.1155/2008/576862

**Published:** 2008-05-25

**Authors:** Dietrich Brandenburg

**Affiliations:** Institute for Clinical Research and Development, D-55116 Mainz, Germany

## Abstract

Starting with the epoch-making discovery of proinsulin, C-peptide has played an important interdisciplinary role, both as part of the single-chain precursor molecule and as an individual entity. In the pioneering years, fundamental systematic experiments unravelled new biochemical mechanisms and chemical structures. After the first detection of C-peptide in human serum, it quickly became a most useful independent indicator of insulin biosynthesis and secretion, finding application in a rapidly growing number of clinical investigations. A prerequisite was the development of specific immuno assays for proinsulin and C-peptide. Further milestones were: the chemical synthesis of several C-peptides and the accomplishments in the synthesis of proinsulin; the detection of preproinsulin with its bearings on understanding protein biosynthesis; the pioneering role of insulin, proinsulin, C-peptide, and mini-C-peptides in the development of recombinant DNA technology; and the discovery of the enzymes for the endoproteolytic processing of proinsulin into insulin and C-peptide, completing the pathway of biosynthesis. Today, C-peptide continues to serve as a special diagnostic tool in Diabetology and related fields. Thus, its passive role is well established. Evidence for its active role in physiology and pathophysiology is more recent and is subject of the following contributions.

## 1. INTRODUCTION

The discovery of proinsulin by Steiner and his coworkers. ([1], and references therein) not only opened the way to understand the mechanism of insulin biosynthesisbut also stimulated the development of the prohormone concept, which was first proposed by Givol in 1965 (see [2]). It was a milestone in the elucidation of the biosynthesis of proteins.

As soon as the structure of proinsulin was known, research developed along two interlinked lines: the intact proinsulin parent molecule, and its “offspring,” the connecting peptide or C-peptide. Proinsulin research proceeded upstream towards the precursor, downstream towards conversion, and in width, directed, for example, at various species. Experimental work took place on all levels, from in vitro studies on the subcellular scale to cells and tissues to in vivo studies in animals. Very soon, the investigations were directed towards man. Studies in healthy man and patients gained increasing importance in diabetes research and clinics. There were two aspects: C-peptide as an integral part of proinsulin, and C-peptide as an individual entity. Soon after the initial biochemical studies C-peptide began its own, more and more independent life. Again, there were two main directions: C-peptide as a diagnostic marker in diabetes mellitus, and C-peptide as a bioactive molecule. These studies, based on the *native* molecule(s), were supplemented by synthetic chemistry.

**Figure 1:**
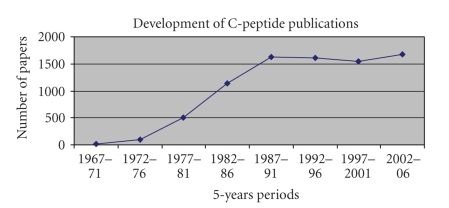
Development of C-peptide publications.

The literature on C-peptide is extensive. Figure 1, based on a search in Medline, depicts the development in 5-year intervals. In view of the large body of publications, this historical review can only be very fragmentary. In particular, while giving credit to individual scientists as far as possible, full references will largely be restricted to selected reviews. Since biological activity of C-peptide is the theme of this special issue, this question will only be tapped.

## 2. THE FIRST DECADE 1967–1976

### 2.1. The pioneering years

The successful chemical synthesis of insulin A- and B-chains, and their combination to give biologically active insulin preparations, was a milestone in peptide/protein science [2]. But the low yields obtainable through combination of the separate chains raised again the crucial question: how is insulin biosynthesis so efficiently accomplished by Nature?

It is now exactly 40 years, that the answer came through the pioneering work of Don Steiner and Philip Oyer [3]. Labelling experiments with a human islet cell adenoma lead to the discovery of a protein of molecular weight 10,800. Upon cleavage with trypsin, it yields insulin-like material. The conclusion is “It is possible that this precursor protein consists of a single polypeptide chain beginning at its N-terminal end with the B chain sequence of insulin, terminating with the A chain sequence, and bearing an additional stretch of polypeptide between the normal chain sequences.”

Subsequent experiments with human tissue and isolated islets from rats elegantly show that the biosynthesis of the precursor precedes the appearance of insulin, and the name “proinsulin” is suggested for this protein [4].

In 1968, Chance et al. [5] isolate and characterise proinsulin from crystalline porcine insulin. They elucidate the amino acid sequence of a 33-peptide, which links the insulin chains and is designated “connecting peptide.” Thus, the findings and concept of Steiner are confirmed and extended, and all future work can now be based on solid protein-chemical grounds. Figure 2 depicts the structure of C-peptide and shows the sequence of human C-peptide.

Systematic followup studies in the laboratory of Steiner et al. [4] quickly shed more light on the exciting findings. Work is in two major directions, connected by many factual and intellectual cross-links. Biochemical investigations on the synthesis in in vitro systems, and isolation/characterisation from available sources. Proinsulin and C-peptide-like compounds are found as by-products of crystalline porcine insulin and in commercial insulin preparations; they can be detected in, and isolated from, human plasma, urine, and pancreas.

The key question directed at the role of C-peptide is answered in an impressive experiment: Steiner and Clark. (1968, see [4]) can successfully demonstrate that fully reduced proinsulin gives, upon reoxidation, high yields of proinsulin. Thus, the primary role of proinsulin appears to be to facilitate efficient formation of the disulfide bonds of insulin.

In the following year, it can be shown that two proinsulins are biosynthesised in the rat. The free connecting segment of proinsulin can be detected and is named C-peptide. It is found that about equivalent amounts of insulin and C-peptide are secreted. C-Peptide can be isolated from bovine and human pancreas, and the sequence of bovine proinsulin is elucidated [4].

A most fruitful collaboration between the Departments of Biochemistry and of Medicine has begun in Chicago, and the biochemical studies are impressively extended towards clinical diabetes research. Already in 1969, a differential immunoassay with proinsulin and insulin antibodies is developed by Rubenstein et al., See [6], and allows the detection of C-peptide in human serum and plasma. The authors consider a possible regulatory function of C-peptide outside of the ß-cell.

On the chemical side, peptide scientists at Hoechst Company succeed already in 1968 in the first chemical synthesis of porcine C-peptide (Geiger et al. [7]). The peptide chain is assembled by synthesis in solution from several fragments.

Subsequently, the emphasis is further shifting towards studies in man. An important prerequisite is the availability of methods for the unequivocal determination of C-peptide. To this end, a specific radioimmunoassay is developed on the basis of isolated human C-peptide and ^131^I Tyr-C-peptide. Equimolar amounts of insulin and C-peptide are found both in pancreas and in the circulation (Melani et al., 1970, see [6]). Extended studies in humans show the presence of C-peptide in the circulation of healthy as well as obese persons and point towards the potential value of C-peptide as independent indicator of beta cell function.

The availability of porcine proinsulin enables Lilly scientists to carry out a number of experiments on the conversion of the prohormone and the chemical as well as biological and immunological characterisation of the resulting intermediates (Chance, 1970, see [8]). First experiments towards crystallisation and preliminary X-ray analyses of proinsulin crystals are reported.

In 1971, the 50th Insulin anniversary is celebrated in a symposium in Indianapolis. It is the appropriate platform to present and discuss the state of the art.

Intensive protein-chemical work of several groups, especially in Chicago and at Novo company in Denmark, on the isolation and analysis of proinsulin and C-peptide results in the preparation of human C-peptide (Markussen et al., 1971) and the determination of its sequence (Oyer et al., 1971, Ko et al., 1971), the isolation of bovine C-peptide (Steiner et al., 1971, Salokangas et al., 1971), and the confirmation of its sequence, as reviewed in [8]. A revisit of the sequence of pork proinsulin leads to a minor correction: Gln5 → Glu.

In refining studies on the proinsulin ⇒ insulin conversion, a useful model for the converting enzyme system of the ß-cell is worked out. The treatment in vitro of bovine proinsulin with trypsin in combination with an excess of carboxypeptidase B leads to intact insulin and C-peptide in an essentially quantitative process (Kemmler et al., 1971, see [9]).

## 3. THE NEXT FIVE YEARS 1972–1976

In the beginning, the primary structures of the C-peptides of several other species are elucidated, as rat, horse, monkey, sheep, and dog in Steiner's laboratory, followed in 1973 by the sequences of duck (Markussen & Sundby), and 1974 of guinea pig C-peptide. In the same time, systematic studies on the conversion of proinsulin to insulin in isolated rat islets and subcelllular fractions are carried out and shed more light on these processes (Kemmler et al., 1973, Tager et al., 1973) For references, see Steiner et al. [9], Kitabchi [10].

In 1975/6, another milestone is set by Don Steiner and his coworkers [11] with fundamental bearings for our understanding of protein biosynthesis. Cell-free translation experiments in islets of rats or islet tumors lead to generation of a labelled protein of molecular mass of 11,500 daltons with 23 additional amino acids NH_2_-terminal to the B-chain sequence of proinsulin, which is named preproinsulin. The N-terminal amino acid sequence of this hydrophobic extension is NH_2_-X-Leu (Lys) Met-x-Phe-Leu-Phe-Leu-Leu (Lys) Leu-Leu-x-leu- (Chan et al. [11]). The signal sequence is rapidly cleaved. Signal peptide extension at/near the N-terminus is later found in almost all secretory proteins (animal, plant, bacteria), see [9].

With respect to the fate and role of C-peptide in the organism, investigations in the rat define the kidney as the main organ responsible for the degradation of proinsulin and C-peptide (Katz and Rubenstein,1973, see [9, 10]). Very careful and extended studies by Kitabchi on the possible physiological function and activity of C-peptide under various conditions are all negative. In conjunction with other studies from the literature, it is concluded that the molecule is devoid of activity [8].

This period is characterised by considerable activities in peptide chemistry. The challenge of a total synthesis of human proinsulin, which in those days meant the assembly of all 86 amino acids in solution, is taken up in Japan and Germany in 1972/3 (see Naithani et al. [12], and Yanaihara et al. [13], and references therein). Several proinsulin peptides are made, and a new synthesis of porcine C-peptide is accomplished, shortly followed by the chemical synthesis of human C-peptide in two laboratories. A considerable number of peptides from human, porcine, and bovine C-peptide are made, mainly for immunological purposes. Modified C-peptides of human proinsulin have improved properties for radioimmunoassay (see below).

The redox experiments of Steiner and Clark on the formation of disulfide bridges between insulin chains had clearly demonstrated the necessity for changing the bimolecular process (as in chain combinations with separate chains) into a monomolecular, intramolecular reaction. However, the important question of chain length remained open. Redox experiments with miniproinsulins give the answer. They demonstrate that a short bridge of only 8 carbon atoms can fully play the role of C-peptide and lead to correct SS pairing in high yield (Brandenburg and Wollmer [14]). These findings are confirmed and extended by Lindsay, Geiger & Obermeier, and Busse & Carpenter, see [15]. In subsequent detailed studies on reduction/reoxidation of proinsulin, Markussen & Heding (1973, 1975, see [16]) determine parameters for bovine proinsulin formation. The studies with miniproinsulins pave the grounds for later insulin production via minimal B-A connection [16].

In parallel to preparative chemistry, physical-chemical studies in several laboratories between 1972–1976 aim at obtaining information on the crystallisability, three-dimensional structure, and conformation of proinsulin/C-peptide (e.g., Pekar and Frank, 1972; Voigt and Wollmer, 1976, see [9, 10]).

Between 1973 and 1976, there is a considerable increase of immunological studies. Chemically synthesised C-peptides, their derivatives, and fragments allow exploring the immunological properties of human, porcine and bovine C-peptide [12, 13], and the generation of various specific antibodies for the development of immunoassays, particularly RIAs, as tools for in vivo studies and analyses under normal/disease conditions as well as tests for antigenicity. Attachment of tyrosine to C-peptide allows later labelling with 125-iodine, and hence synthetic human ^125^I-Tyr-C-peptide [12] plays a special role as tailor-made tracer molecule. An N-terminal derivative, human carbobenzoxy (Z)-C-peptide, gives, after immunisation with albumin-conjugated antigen, particularly high titers in guinea pigs [17].

With the available tools of RIA, the number and extent of experimental in vivo investigations and particularly of clinical studies is rising. C-Peptide assays are used in a large variety of studies, for example: on liver metabolism, on ketoacidosis, in looking at infants of insulin-treated diabetic mothers, in insulin-induced hypoglycemia, during oral glucose tolerance test, or in comparing portal and peripheral blood. Further studies extend to patients with islet cell tumors or to the stimulation by OADs. Examining children with juvenile diabetes, Ludvigsson and Heding [18] find that of 96 diabetic children, 35.4% had detectable levels of C-peptide. For a detailed discussion of the clinical significance of circulating proinsulin and C-peptide see Rubenstein et al. [19].

## 4. THE SECOND DECADE 1977–1986

By 1977, major goals of fundamental research have already been accomplished. For reviews, see Steiner “Insulin today” [20] and Kitabchi [10]. C-peptide is now becoming an ever growing factor in clinical research and diagnosis under various conditions.

As seen in Figure 1, there is a steep rise in the number of publications describing work concerned with C-peptide. For the year 1977, PubMed yields 72 answers to “C-peptide + proinsulin,” of which about 43 = 60% concern a large variety of studies which are based on the determination of C-peptide in blood, plasma, or urine. Thus, besides limited further work of a more fundamental nature, studies are almost exclusively application directed, that is, making use of the unique properties of C-peptide as an independent indicator of beta cell function and insulin secretion under normal and diabetic conditions.

The state of the art at the beginning of this decade is summarised at three congresses: In 1978 the C-peptide symposium in the USA and the symposium on proinsulin, insulin, and C-peptide in Tokushima, Japan, followed in 1979 by the international symposium on insulin and related hormones in Aachen, Germany. Experimental work in chemistry and biochemistry has well advanced and leads to remarkable achievements: the total chemical synthesis of complete proinsulin from 16 fragments has been accomplished in Japan, yielding a product with 10% immunoreactivity (Yanaihara et al., 1979, see [21]). The German competitors in Aachen succeed in synthesising the two fragments 1–45 and 46–86, spanning the whole sequence of the prohormone [21]).

In biochemistry and molecular biology, the studies on the proinsulin precursor succeed, in 1977, in the isolation and characterisation of bovine preproinsulin from a cell-free translation system and in 1978 in the detection and identification of preproinsulin in pancreatic islets (see [22]).

Again a new chapter of extreme importance and consequences is being opened: recombinant DNA technology. As in several cases before, insulin is assuming a pioneering role. Although a two-chain molecule, it is the first candidate to be built up in *E. coli* via this revolutionary approach. First detection of immunoreactive insulin [23] is soon followed on the preparative scale [24]. While the approach via the separate A- and B-chains and subsequent combination is surprisingly efficient, the proinsulin gene is the optimal precursor [25]. Thus, besides human insulin for diabetes treatment, human proinsulin and C-peptide become now available in quantity for research and other applications. Only two years later, the processes are successfully transferred to eucariontic cells (Lomedico et al., 1982, see [1]).

The application of C-peptide as a diagnostic tool depends critically on the availability of sensitive and selective analytical methods. Although by 1976 progress has been marked, the need for further development and refinement of immunological methods, in particular RIA, is considerable. It is interesting to note that in 1977 not less than 5 papers on the development of RIAs are published in Japan. Besides other problems, the scarcity of antigen probes certainly is a limiting factor. Since 1980, this obstacle is overcome through the availability of biosynthetic human proinsulin and C-peptide. Examples for immunological studies are a very careful study with three assay systems using different antisera by Kuzuya et al., 1978, see [26], aiming at the detection and avoidance of pitfalls. Human proinsulin-specific antigenic determinants can be identified by monoclonal antibodies and allow hints towards the conformation of C-peptide (Madsen et al., 1984, see [27]).

The advances in methodology as well as the results of the large number of clinical studies between 1980 and 1986 are compiled in three reviews by Gerbitz [26], Polonsky and Rubenstein [27] as well as Faber and Binder [28]. Pitfalls and limitations in the determination of the secretion and hepatic extraction of insulin are discussed in [27]. The use of peripheral C-peptide concentrations is considered as a valuable *semiquantitative* marker of beta cell secretory activity. However, accurate quantification is more difficult and not yet possible. Confirming earlier observations, it is stated that as many as 15% of patients with type-1 diabetes retain life-long beta cell function that persists at approximately 10% of that observed in nondiabetic individuals [28].

## 5. THE THIRD DECADE 1987–1996

The investigations of the mechanism of biosynthesis lead to another success. In 1990/1991, after long, extended search in several laboratories, the enzymes responsible for the endoproteolytic processing of proinsulin into insulin and C-peptide are finally discovered. PC2, a 638-residue protein is identified via a human insulinoma DNA by Smeekens and Steiner (1990), and PC3 (PC1), a 753-residue protein, by Smeekens et al., 1991. Both are serine proteases related to subtilisin. After selective cleavage at the C/A junction Lys-Arg or the B/C junction Arg-Arg, products with C-terminal basic residues are generated. These residues are, as shown later, finally removed by carboxypeptidase E, an exopeptidase. For a detailed discussion, see Steiner [1, 29].

Clinical aspects of C-peptide as a diagnostic tool remain in the centre, as documented by a large number of publications. For example, C-peptide is a valuable parameter in the assessment of beta cell function in pancreas transplant recipients, as reported by a Danish-Swedish Study Group in 1994 [30].

Since 1994, the question “does C-peptide have a physiological role?” gains grounds and finds positive answers in several directions [31]. By 1996, positive experimental evidence is hardening, as new physiological effects are investigated. The conclusion is: C-peptide is a biologically active hormone [32]. A negative aspect is the observation that in vitro studies point towards a possible involvement of C-peptide in the formation of amyloid-like fibrils. It thus may be of importance in the pathogenesis of amyloid in the islets of Langerhans [33].

## 6. THE FOURTH DECADE 1997–2006

A very vivid portrait of C-peptide is painted by Don Steiner in his review “The proinsulin C-peptide—a multirole model” [1]. It summarises our knowledge from evolutionary aspects to the role of C-peptide in processing from its structure to analysis and clinical application. The latter comprises, as before, the bulk of published studies in this period.

A very substantial account of assays for insulin, proinsulin(s), and C-peptide available in 1999 is given by Clark [34]. Further methodological advances aim now at overcoming previous difficulties in accounting for the large differences in metabolic clearance of C-peptide versus insulin. Newly developed effective methods for deconvolution of C-peptide values allow widespread use of C-peptide assays for evaluation of ß-cell function (Polonsky and O’Meara, 2001, see [1]). Labelling with stable isotopes is proposed as a new tool for in vivo pharmacokinetic and metabolic studies [35]. The importance of C-peptide in the classification of diabetes mellitus, as well as its potential clinical applications, is reviewed on the basis of a Medline literature search [36].

More studies are directed towards the biological activity of C-peptide. Based on observations with pancreas transplants, the combined replacement of insulin and C-peptide in diabetes treatment is deemed beneficial [36]. Two international symposia gather the experts and summarise the state of the art: in 2000, “*Cellular, physiological and clinical effects of C-peptide*” in Detroit, Michigan, and in 2003 “*Physiological and pathophysiological activities of C-peptide.*” Beyond the presentation of research results, the meetings are aiming at the potential therapeutic value of C-peptide replacement in preventing and ameliorating type 1 diabetic complications and at identifying the immediate directions of C-peptide research (see [37] and subsequent papers).

## 7. THE FIFTH DECADE 2007

At the beginning of the fifth decade, clinical applications continue to be the predominant field; there is no new work in chemistry or physical chemistry. C-peptide measurements are compared in an international action in which 15 laboratories from 7 countries participate [38].

A very active area is stem cell research. C-peptide is again a very useful marker of cell activity in exploring systems and culture conditions. It has particular importance as an independent indicator, because the source of insulin under the conditions of cell culture is not always unequivocal [39]. Besides several studies with human or murine embryonic stem cells, mesenchymal stem cells were investigated, for example in [40]. Human umbilical cord blood-derived stem cells can be engineered to engage in de novo synthesis of insulin, as first demonstrated by Denner et al. [41]. C-peptide is also a good independent marker for insulin synthesis under the conditions of beta cell culture [42].

As demonstrated by insulin and C-peptide secretions, beta cells occur naturally in extrahepatic bile ducts of mice (Dutton et al. [43]). This discovery has significance both with respect to evolution as well as for regenerative medicine, pointing towards a new source of beta cells.

With respect to biological activity, new studies are reported from John Wahren's laboratory. Based on internalisation experiments, it is concluded that C-peptide has properties of an intracrine peptide hormone (Lindahl et al., see [44]. C-peptide treatment for 6 months improves sensory nerve function in early-stage type 1 diabetic neuropathy, as found in a study with 139 patients (Ekberg et al., see [44]). The present stand is summarised by Wahren [44] “C-peptide is a bioactive peptide.”

In clinical investigations, C-peptide serves as indicator for beta cell function under therapy with an immunogenic peptide DiaPep277 from the 60-kDa heat-shock protein [45], or treatment with thiazolidinediones, in testing the effects of dipeptidyl peptidase-4 inhibitors [46] and in a large study on the incidence of diabetes in 2435 youths in the United States [47]. Increased C-peptide levels, in conjunction with other parameters, are found to be valuable indicators of risk of colorectal cancer, see, for example, [48].

## 8. CONCLUSIONS

The history of proinsulin and C-peptide has been coined and now over 40 years been intimately linked with their discoverer, Don Steiner. The splendid original research by him and his associates has been supplemented by excellent reviews, which accompanied the experimental work and reflected the state of the art as a red tape. Towards the end of 4th decade, in 2005, Don Steiner was honoured by a symposium on the occasion of his 75th birthday “Exploring Pancreatic Beta Cell, Insulin Biology and Protein Processing.” It gathered friends and researchers from all over the world, and the programme of the Don Steiner Fest, held at the University of Chicago, mirrored the scientific avalanche triggered in 1967.

Based on exciting fundamental science, and beyond its role as an integral part of proinsulin, C-peptide has quickly found rising attention. The reason is its unique property as an independent marker of insulin biosynthesis and secretion, which makes it a special diagnostic tool in Diabetology and related fields.

Progress depends on ideas, suitable methods, and adequate tools. Such a tool is C-peptide. Proof of significance is simple and becomes obvious from the mere quantitative look at the literature—the annual number of scientific publications (see Figure 1). The steep rise beginning only 10 years after its discovery results in over 200 papers in 1984, and from 1988 on to more than 300 per year.

While this *passive* role as diagnostic tool is now established for a rather long time, the *active* role in physiology and pathophysiology is more recent, and is a subject of current and future research. This will be reflected in the current issue.

## Figures and Tables

**Figure 2:**